# Spontaneous umbilical endometriosis

**DOI:** 10.1093/skinhd/vzaf044

**Published:** 2025-06-25

**Authors:** Vasilina Tambovskaya, Luis Escalante, Astrid Maldonado, Maria Antonieta Touriz Bonifaz, Edgar Escalante

**Affiliations:** Department of Dermatology, Hospital IESS Quito Sur, Quito, Ecuador; Department of Dermatology, Universidad de Especialidades Espiritu Santo, Samborondon, Guayas, Ecuador; Department of Dermatology, Hospital Solca Núcleo de Tungurahua, Ambato, Tungurahua, Ecuador; Department of Dermatology, Universidad de Guayaquil, Guayaquil, Guayas, Ecuador; Department of Dermatology, Ephora Research Group, Guayaquil, Ecuador; Department of Dermatology, Universidad de Guayaquil, Guayaquil, Guayas, Ecuador; Department of Dermatology, Universidad Católica de Santiago de Guayaquil, Guayaquil, Guayas, Ecuador; Department of Dermatology, Universidad de Guayaquil, Guayaquil, Guayas, Ecuador; Department of Otorhinolaryngology, Hospital Carlos Andrade Marin, Quito, Ecuador

## Abstract

Endometriosis is defined as the presence of endometrial tissue (glands and stroma) outside the uterine cavity. Various organs can be affected, including the skin. Umbilical endometriosis is the most common clinical form of extrapelvic endometriosis and the most frequent type of cutaneous endometriosis, classified into primary and secondary forms. The primary or spontaneous form is rare, occurring without any prior surgical intervention, with a predilection for the umbilical area.

A 30-year-old woman presented with a 6-month history of a painful umbilical nodule that exhibited cyclical bleeding corresponding to her menstrual cycle. The lesion was firm, brownish-grey, and measured 0.4–1 cm in diameter. No prior history of abdominal or pelvic surgery was reported (Figure 1a).

**Figure 1:**
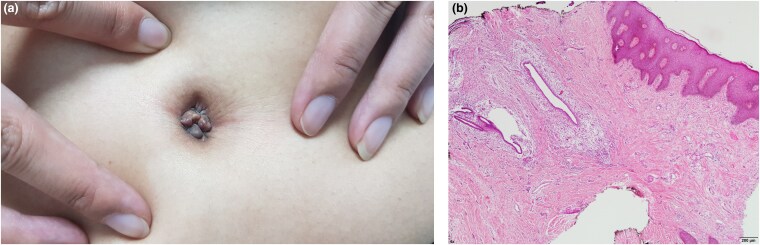
(a) A well-defined, multilobulated, firm, smooth-surfaced umbilical nodule measuring 0.4–1 cm in diameter with a brownish-grey hue. The lesion was tender upon palpation and displayed cyclical changes corresponding to the patient’s menstrual cycle. (b) Histopathology of the lesion reveals endometrial glands and stroma within the dermis, surrounded by a dense lymphocytic infiltrate and siderophages. The dilated glandular structures are lined with cylindrical epithelium, with erythrocyte extravasation, confirming the diagnosis of umbilical endometriosis.

A soft-tissue ultrasound was performed, revealing a well-defined, hypoechoic subcutaneous lesion with no deep extension. Surgical excision under local anesthesia was performed without prior biopsy, given the small size and well-circumscribed nature of the nodule.

Histopathological examination confirmed the presence of ectopic endometrial tissue with characteristic endometrial glands and stroma. The findings were consistent with spontaneous umbilical endometriosis, a rare form of extrapelvic endometriosis (Figure 1b).

The patient was referred to a gynecologist for further evaluation. Pelvic imaging and clinical assessment showed no other endometriotic foci. At 6-month follow-up, she remained asymptomatic with no recurrence.

Umbilical endometriosis, also known as Villar’s nodule, is the most common type of cutaneous endometriosis and may occur spontaneously or secondary to surgical intervention. Primary umbilical endometriosis is a rare entity, with an incidence of 0.5–1% among ectopic endometrial cases.^1,2^ The lesion may present as a reddish-blue or brown nodule, often associated with premenstrual or menstrual pain.

Surgical excision remains the gold standard for diagnosis and treatment. Imaging, such as ultrasound or magnetic resonance imaging, may help assess lesion extent. Women presenting with painful umbilical nodules should be evaluated for possible endometriosis.

**Funding sources:** This research received no specific grant from any funding agency in the public, commercial or not-for-profit sectors.

**Data availability:** No data generated.

**Ethics statement:** The study adheres to the ethical guidelines for case reports and image submissions.

**Patient consent:** Informed patient consent for publication, including for use in social media, has been obtained.
